# Climatic Impact of Volcanic Eruptions

**DOI:** 10.1100/tsw.2002.83

**Published:** 2002-04-03

**Authors:** Gregory A. Zielinski

**Affiliations:** Institute for Quaternary and Climate Studies and Department of Geological Sciences, University of Maine, Orono, ME 04469, USA

**Keywords:** volcanism, climate, climatic change, atmospheric aerosols

## Abstract

Volcanic eruptions have the potential to force global climate, provided they are explosive enough to emit at least 1–5 megaton of sulfur gases into the stratosphere. The sulfuric acid produced during oxidation of these gases will both absorb and reflect incoming solar radiation, thus warming the stratosphere and cooling the Earth’s surface. Maximum global cooling on the order of 0.2–0.3°C, using instrumental temperature records, occurs in the first 2 years after the eruption, with lesser cooling possibly up to the 4th year. Equatorial eruptions are able to affect global climate, whereas mid- to high-latitude events will impact the hemisphere of origin. However, regional responses may differ, including the possibility of winter warming following certain eruptions. Also, El Niño warming may override the cooling induced by volcanic activity. Evaluation of different style eruptions as well as of multiple eruptions closely spaced in time beyond the instrumental record is attained through the analysis of ice-core, tree-ring, and geologic records. Using these data in conjunction with climate proxy data indicates that multiple eruptions may force climate on decadal time scales, as appears to have occurred during the Little Ice Age (i.e., roughly AD 1400s–1800s). The Toba mega-eruption of ~75,000 years ago may have injected extremely large amounts of material into the stratosphere that remained aloft for up to about 7 years. This scenario could lead to the initiation of feedback mechanisms within the climate system, such as cooling of sea-surface temperatures. These interacting mechanisms following a mega-eruption may cool climate on centennial time scales.

## INTRODUCTION

During the summer of AD 1783, Benjamin Franklin, then the U.S. ambassador to France, noted that there was a dry fog across Europe that did not dissipate even after it rained ([1] as reprinted in[2]). He thus suggested that this was not the typical fog associated with common meteorological conditions. Furthermore, he noted that the weather was rather cool and overall quite different compared to other years. He concluded that the cause for these conditions must have been the ongoing volcanic eruption of Lakagigar (Laki for short) in Iceland. It was this observation by Franklin that caught the attention of modern-day scientists, and especially climatologists, interested in identifying the factors that cause the Earth's climate to change.

The significant impact of volcanism on climate became more apparent in the early AD 1800s, following the very large eruption of Tambora (Sumatra, Indonesia) in AD 1815[3]. The summer of AD 1816 is now known for the exceptionally cool weather observed throughout the Northern Hemisphere[4]. In fact, several noteworthy events characterize the climatic conditions of 1816[3,4]. These include June snow in parts of northern and western New England in the U.S. as well as July and August frosts in these same areas. In addition, a very cold summer characterized much of Europe, leading to widespread crop failures and famine. The cool summer did not seem to be as prevalent in Asia, although, interestingly, the summer of AD 1783 in Japan was especially cool and wet. These abnormal weather and climatic events in North America and Europe ultimately led to AD 1816 being referred to as the "Year Without a Summer." Interestingly, the cool weather of AD 1816 was the culmination of several years with overall cool to cold conditions that appeared to begin about AD 1809. There is evidence from marine temperature records[5] and ice-core data[6,7] of a significant volcanic eruption late in AD 1808 that may have been a major contributor to the initial cooling in the AD 1810s, but the eruption has not been specifically identified. The significance of ice-core research in understanding the volcanism-climate system[8] is discussed later. Thus, a major portion of this significant cool period in the early AD 1800s may have been a function of explosive volcanic activity.

These two examples give a brief introduction into how it initially became known over the last 200 years that volcanic eruptions have an impact on the Earth's climate. However, our understanding of how volcanism forces climate is much more detailed now, given the existence of instrumental climatic records, high-technology instrumentation including satellites, and other techniques that have opened up the door to understand better the volcanism-climate system. This review summarizes our knowledge to date of this dynamic part of the Earth's environment, although there is still much to learn given that instrumental records exist for only the last 100 years to occasionally 200 years and high technology has been available for only the last 20–30 years. The next section focuses on why particular volcanic eruptions can have a large impact on climate, while other explosive eruptions have only a minimal impact. Similarly, some eruptions have only a hemispheric impact, whereas others will have a global impact. The third section discusses the magnitude of the impact based on instrumental temperature records, that is, the known impact over the last 100–200 years. However, the number and type of volcanic eruptions observed over the last few centuries are small when considering the potential range of eruption size and type that have occurred in the geologic record. Consequently, to understand better the complete volcanism-climate system, proxy records of the impact of past volcanism need to be used. These techniques and the results they provide also are discussed. Finally, the potential impact of multiple eruptions closely spaced in time and of mega-eruptions, eruptions that have not occurred in modern history, is discussed. General conclusions comprise the final section of this review.

This summary approaches the subject of the volcanism-climate system from both a climatological and geological perspective. Other reviews have a different emphasis, including approaches more from an atmospheric science or modeling perspective. Individuals are encouraged to read the following review articles, and references therein, to obtain a more complete understanding of the impact volcanic eruptions have on climate. Other reviews include those by Lamb[9], Toon[10], Ellsaeser[11], Kondratyev and Galindo[12], Robock[13], and Zielinski[8]. Two other articles provide an excellent review of not only the climatic impact of a volcanic eruption but also the overall atmospheric perturbation caused by the eruption. These articles use examples from the two recent eruptions of El Chichón (Mexico, 1982)[14] and Pinatubo (Philippines, AD 1991)[15] as a means of summarizing the overall atmospheric impact of a major eruption.

## HOW A VOLCANIC ERUPTION IMPACTS CLIMATE

There are two main parameters that determine whether or not a particular eruption will have an impact on climate. One parameter is the nature of the products emitted, including the total volume emitted during the entire eruption sequence and not just during the cataclysmic event. The second parameter of importance is the explosivity of the eruption (i.e., its intensity). These two aspects are discussed in the first two subsections below. Should an eruption satisfy the requirements necessary for it to have an impact on climate, there remains an additional parameter that determines the spatial extent of this impact (i.e., will the impact affect the entire globe or only a portion of the globe). Location of the volcano determines how widespread the impact will be, as detailed in the third subsection below.

### Products of an Eruption

The material injected into the atmosphere by a volcanic eruption has the ability to absorb and reflect incoming solar radiation (Fig. 1). Essentially, the solar radiation encountering this material is scattered as opposed to passing directly to the Earth's surface. This process will heat the layer of the atmosphere in which these particles or aerosols reside, while at the same time preventing some of the incoming solar radiation from reaching the Earth's surface. The ultimate result is that a volcanic eruption can cool the Earth's climate. However, the different characteristics of the ejected material produce different results as to the ability to scatter solar radiation.

When a volcano erupts, it spews out two main products. One component is the mineral or silicate matter that produces widespread ash deposits or lava, depending on the type of eruption. The term *ash* is used collectively here to describe the ejecta produced during the explosive phase of an eruption, although ash is defined as a grain size for volcanic deposits. *Tephra* often is used as an all-encompassing term for airfall deposits. The effusive phase of an eruption predominantly produces lava. Although there are large quantities of ash produced in a major eruption, the climatic impact of this eruptive product is minimal both in space and time. This limited impact results from the large size of this material, thus it cannot remain aloft for a long period of time and it cannot travel far from the erupting vent in sufficient enough quantities to have a widespread impact on climate. Although individual grains are very efficient in absorbing and reflecting incoming solar radiation, more so than the other volcanic products[16], their short life in the atmosphere produces a limited climatic impact. Nevertheless, this impact can be significant in the areas close to the source volcano, at least for a short period of time, as was evident for areas in the northwestern U.S. following the AD 1980 Mt. St. Helens eruption[17].

The second component emitted during the eruption, as well as during the quiescent phase prior to the climactic eruption, is volcanic gas. The most abundant gases released include such elements as sulfur, carbon, chloride, and fluoride. Water vapor also is an abundant gas released during an eruption. Once these gases are injected into the atmosphere, they react with other compounds (such as water), possibly forming acid particles or aerosols like sulfuric acid ($H_2SO_4$) and hydrochloric acid (HCl). These acidic aerosols often are composed of both acid and water, often at a ratio of 75% acid and 25% water[18]. The acidic droplets are lighter than the ash particles injected into the atmosphere, thus the acidic aerosols can remain aloft in large quantities for much longer periods of time than the ash. Aerosols that form in the stratosphere may remain there in a sufficient enough quantity to impact climate for 3–4 years following the eruption. Those aerosols that form in the troposphere, where the Earth's weather occurs, will be washed out by precipitation in a very short period of time, that is, on the order of a week or two.

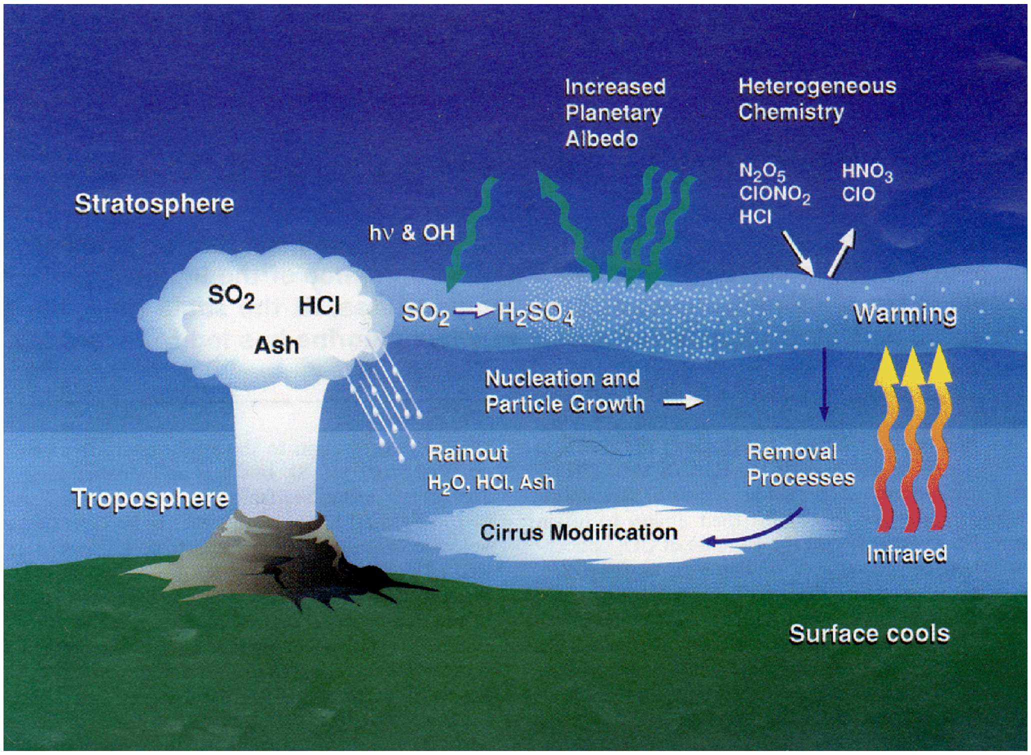

**FIGURE 1.** Schematic diagram showing the products injected into the atmosphere following an explosive volcanic eruption, chemical and physical processes that may occur and the potential climatic response. See text for details. (After McCormick, M.P., Thomason, L.W., and Trepte, C.R. Atmospheric effects of the Mt. Pinatubo eruption. *Nature* **373**, 399–404, 1995.) With permission from *Nature* (http://www.nature.com).

The type of acid that is primarily responsible for absorbing or reflecting incoming solar radiation, and thus cooling the Earth's climate, is $H_2SO_4$ (Fig. 1). The two types of gases emitted during an eruption that eventually form the $H_2SO_4$ in the atmosphere are $SO_2$ and $H_2S$, depending on the composition of the original magma in the volcano's plumbing system. However, any $H_2S$ emitted often oxidizes quickly to $SO_2$. Similarly, the $SO_2$ injected into the atmosphere completely converts to $H_2SO_4$ within about a month following the eruption, as was observed following the 1991 Pinatubo eruption. Satellites carrying the total ozone mapping spectrometer originally designed to measure ozone loss were found to identify and measure $SO_2$ concentrations in the stratosphere[19]. Many of the other acids (like HCl and hydrofluoric acid, HF) are much more soluble and will not stay aloft for a long period of time. A great proportion of these other acidic aerosols often will adsorb onto ash particles within the eruption column and fall out of the atmosphere very quickly[20,21] (Fig. 1). However, some HCl and HF can remain aloft for a long period of time while at the same time traveling far from the eruptive vent, but they will not be in the quantities needed to impact climate[22,23]. $H_2SO_4$, on the other hand, will remain in the stratosphere in sufficient enough quantities to cool the Earth's climate. Consequently, a volcanic eruption needs to release large amounts of sulfur for it to be a "climatically effective" eruption.

Given that an eruption needs to emit large quantities of sulfur to have an impact on climate, the follow-up question is, how much sulfur is needed? The critical parameter that determines how much sulfur is needed to impact climate is the optical depth of the stratosphere. The optical depth ($\tau$) is a value that essentially determines how much solar radiation reaching the top of the Earth's atmosphere is able to reach the Earth's surface. The higher the $\tau$ value, the less solar radiation that reaches the surface. It appears that to have observable cooling at the Earth's surface following an eruption, the optical depth of the stratosphere needs to be about 0.1[24]. By using the relationship between optical depth ($\tau_D$) and mass of the total $H_2SO_4$ aerosol loading ($M_D$), where $\tau_D = M_D/1.5 \times 10^{14}$ g[25], it appears that the mass of $H_2SO_4$ needs to be about $10^{13}$ g or 10 megatons (Mt). The mass of gaseous $H_2SO_4$ eventually produced in the stratosphere is about double the amount of $SO_2$ emitted, whereas the mass of the total $H_2SO_4$ aerosol eventually produced in the stratosphere is approximately another 1.25 times the mass of $H_2SO_4$ produced. For example, an eruption that releases 5 Mt of $SO_2$ into the stratosphere will produce about 10 Mt of $H_2SO_4$ and about 12.5 Mt of $H_2SO_4$ aerosol. The calculation to determine the total $H_2SO_4$ aerosol produced is based on an aerosol composition of about 75% $H_2SO_4$ and 25% $H_2O$[18]. Using these calculations and a time series of volcanic $SO_2$ emissions over the last 25 years[26], it has been shown that for an eruption to be climatically effective (such as Pinatubo and El Chichón), it needs to inject at least 1–5 Mt $SO_2$ into the stratosphere. Eruptions producing less than this amount will lose too many aerosol particles too quickly to be climatically effective. Particles settle out of the stratosphere by gravitational settling, that is, with an *e*-folding decay time of about 8–12 months depending on particle size[14]. The decay time is essentially the slope of a best-fit line showing the change in concentration of particles with a specific radius in the atmosphere with time.

## Importance of an Eruption's Explosiveness

The importance of an eruption's explosiveness has been alluded to in the preceding section regarding the ability of $H_2SO_4$ aerosols to remain aloft for a long enough period of time in quantities large enough to cool climate. Simply stated, the eruption must be able to penetrate the tropopause, thereby reaching the stratosphere. Those remaining in the troposphere will be washed out by precipitation. Those remaining in the stratosphere will settle out much more slowly by gravitational settling. These stratospheric aerosols are the key for an eruption to have an impact on climate.

The type of eruption that most readily injects debris into the stratosphere is the very explosive plinian eruption, that is, the type of eruption that produces a large mushroom-like column. These eruptions are most common along subduction zones adjacent to continental margins. However, many of the largest plinian eruptions will develop extensive pyroclastic flows with the collapse of the plinian column because of the increased density of the column associated with a very high eruption rate. These pyroclastic flows move down the slopes of the volcanoes at great speed, ultimately spreading outward over the entire landscape beyond the erupting volcano. Despite this process, very buoyant coignimbrite clouds are produced from these pyroclastic flows, clouds that may reach heights similar to the plinian phase of an eruption (for example, Fig. 3 in[27]). On the other hand, effusive eruptions (i.e., eruptions that produce extensive lava flows like in Hawaii) are less likely to penetrate the tropopause. There are exceptions, however. Very large fire fountains associated with some effusive eruptions can produce very large eruptive clouds that are of sufficient buoyancy to rise into and penetrate the tropopause[28,29].

Two more factors that must be taken into account as to the ability for a particular eruption to penetrate the tropopause are the location of the volcano and the time of year of the eruption. Less explosive eruptions originating from volcanoes in the mid- to high latitudes of each hemisphere (e.g., Iceland, Alaska, Kamchatka in the Northern Hemisphere and Antarctica in the Southern Hemisphere) may penetrate the tropopause if their columns rise to around 10 km. If the eruption occurs in winter, the column needs to reach only about 8 km in height to get into the stratosphere. An equatorial eruption needs to reach about 15 km to penetrate the tropopause regardless of the season. Thus, a smaller eruption can impact climate if it is located in the mid- to high latitudes.

There is an interesting enigma in the sulfur content-explosiveness relationship and the ability for an eruption to impact climate. Very explosive eruptions usually form in volcanic systems with a magma concentration that is high in silica, but often low in sulfur. Thus, not all very large, explosive eruptions will have a significant climatic impact. The AD 1980 Mt. St. Helens eruption is a prime example, as it was very explosive, but had very little if any impact on global climate because of its low sulfur content. In contrast, effusive eruptions commonly occur in volcanic systems with low-silica magma. These magma types can be very high in sulfur. Consequently, an effusive eruption that can produce large fire fountains and buoyant eruption clouds that penetrate the tropopause are able to have a significant impact on climate. The Icelandic Laki (AD 1783) and Eldgjá (~AD 934–938) eruptions are excellent examples of events dominated by the formation of extensive lava flows that, at the same time, were able to place large amounts of sulfur (possibly around 220 Mt[30]) into the stratosphere. Adding to the complexity of the system, large plinian eruptions, like Pinatubo (1991), can have a major impact on climate despite the overall low-sulfur content of their magma. Two possibilities may explain this situation. One possibility is that the very large volume of the eruption compensates for a low (sulfur content/volume magma erupted) ratio. The second possibility is that there is some degassing of the volcanic system prior to the cataclysmic event[31] that led to a high amount of atmospheric sulfur despite the low-sulfur content of the magma, as determined through analysis of the eruptive products. These examples reflect the complexity within the volcanic part of the system. Although it may be easy to say that an eruption will impact climate if it is high in sulfur and if the eruptive column penetrates the tropopause, one cannot always predict accurately the specific eruptions that will or have been climatically effective without a thorough evaluation of the volcanic system in question.

## Spatial Extent of the Impact

The location of an eruption not only will help determine whether or not the eruption column is able to penetrate the tropopause but also is critical in determining the spatial extent of any climatic impact. For an eruption to impact global climate, it must occur in the equatorial zone. The overall distribution of energy via air flow from equatorial regions to the poles carries the volcanic aerosols poleward. Following an equatorial eruption, the aerosols will then expand into each hemisphere as a function of season. There is a greater exchange of air between the tropical and polar regions in the transition seasons of spring and fall, thus they will be the times of year when most of the volcanic aerosols will be distributed into each hemisphere. Poleward migration of aerosols will be quicker following a spring or autumn eruption, whereas there will be a slower distribution when the eruption occurs in the winter or summer. The timing of the climatic impact from an eruption in the mid- to high latitudes will thus depend on the season of the eruption. Furthermore, the distribution into each hemisphere is not necessarily symmetrical, as in addition to the season of the eruption, the location of the intertropical convergence zone and the quasi-biennial oscillation can influence the poleward distribution[8]. For instance, the distribution of aerosols following the three largest most recent climatically effective eruptions in equatorial zones all had a different dispersal pattern. Approximately two thirds of the aerosols produced from the AD March 1963 eruption of Agung (Bali, 8˚S) spread into the Southern Hemisphere, whereas the distribution of the AD June 1991 Pinatubo (Philippines, 15˚N) eruption was symmetrical between hemispheres[32]. The AD April 1982 El Chichón (Mexico, 17˚N) eruption had a majority of its aerosols move into the Northern Hemisphere[14].

Eruptions that occur in mid- or high latitudes will have an impact on the hemisphere in which the eruption originates and not on global climate (e.g.,[33]). There is very little mixing of air masses across the equator, thereby limiting the mass of aerosols that could migrate into the opposite hemisphere. Some aerosols may cross into the other hemisphere, but there probably would not be enough to have a noticeable impact on climate in that hemisphere. On the other hand, the climatic impact will be felt more quickly in the mid- to high latitudes where the eruption occurred than would be felt from a tropical eruption, since the aerosols must migrate into the individual hemisphere. The more-limited global impact from nonequatorial eruptions does not mean that mid- to high-latitude eruptions are unimportant in the overall volcanism-climate system, as they can have a severe impact on the hemisphere of origin. From a human perspective, any large, explosive Northern Hemisphere eruption in the future, as has occurred in Kamchatka and Alaska, would have a very severe impact on the large population centers and agricultural areas of the Northern Hemisphere[8].

## MAGNITUDE OF THE IMPACT

Having established the eruption parameters needed for a particular eruption to have an impact on climate, the next step is to identify exactly what that impact is. Quantifying the impact has been done for those eruptions that occurred during the period when instrumental temperature records are available as presented in the following subsection. However, these evaluations are primarily following a single eruption. To better understand the complete volcanism-climate system, it is necessary to evaluate the climatic impact of eruptions that occurred prior to the instrumental record. To do this, other types of information are essential when available in paleoclimatic and paleoenvironmental records that indicate past climatic conditions (i.e., proxy records). In addition, the record of observed volcanic activity goes back only about 2000 years, with the AD 79 Vesuvius (Italy) eruption being the last observed large eruption. Prehistorical volcanic records must be used in conjunction with climate proxy records to evaluate the many different scenarios (i.e., different types of eruptions and changing climatic modes) possible in the volcanism-climate system. Looking at these records also allows evaluation of the impact of multiple eruptions closely spaced in time and the impact of mega-eruptions, the exceptionally large eruptions that have not been observed in historical time.

### Using Instrumental Temperature Records

Direct evidence that volcanic eruptions cool climate is shown by the lower temperatures in Northern Hemisphere instrumental records over the last 200+ years following almost all major eruptions. This conclusion comes from the time series of temperatures from New Haven, CT, northern and central Europe, and central England compared to the timing of all major eruptions since AD 1740 ([34] and Fig. 2). In nearly every case, there were cool years below the long-term mean following the eruption or a cooling below that of the preceding few years. This becomes even more apparent when the Northern Hemisphere is viewed as a whole (Fig. 2). However, there are other forcing factors at work during this time period, as, in some cases, there are cooler pre-existing conditions for some of these eruptions. This suggests that certain eruptions over the last few centuries may have enhanced and possibly extended the cool climate existing at the time of the eruption.

Quantification of the climatic impact of many of these same eruptions across various latitudinal bands has also been undertaken[35]. Using composites from a series of eruptions in the last 2 centuries shows that the global cooling from a major explosive eruption may be 0.2–0.3˚C in the year of the eruption or in the first year after the eruption (Fig. 2). The complete impact may last for up to 4 years (Fig. 2), with the magnitude of the annual cooling becoming less in each subsequent year following the peak period of cooling.

The most recent evidence of the amount and longevity of cooling following an eruption comes from an analysis of the forcing potential of the aerosols produced by the AD 1991 Pinatubo eruption as compared to that for other forcing factors[15]. Pinatubo aerosols produced a forcing of –2.4 W/m$^2$ in AD August 1991, > –3 W/m$^2$ in AD August 1992, and then ~ –1 W/m$^2$ in AD August 1993, 2 years after the eruption. The combined forcing component of all greenhouse gases over this time period was about +2.2 W/m$^2$ with solar irradiance accounting for another +0.3 W/m$^2$. Thus, the climatic-forcing potential of the Pinatubo aerosols was greater than or equal to these other forcing mechanisms (i.e., warming mechanisms) during the first 2 years following the event. Interestingly, the Pinatubo eruption is only moderate in size from a geological perspective.

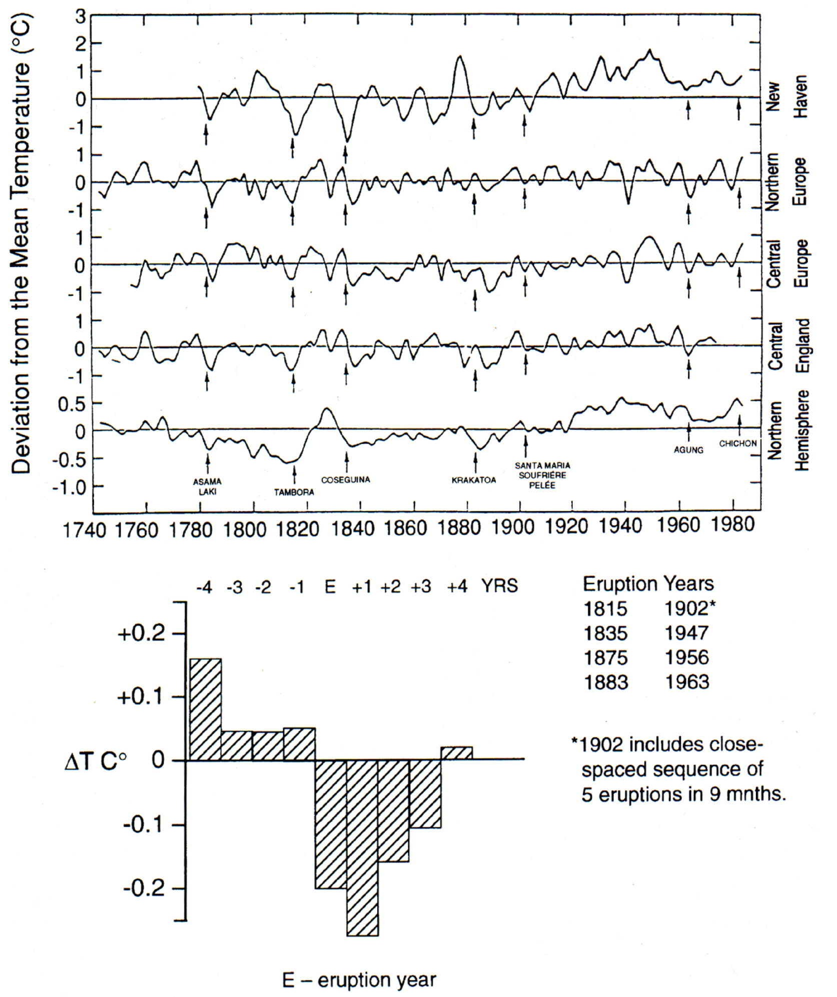

**FIGURE 2.** Northern hemisphere temperature trends (˚C) as related to volcanic eruptions over the last 260 years (top). Temperature are smoothed primarily through a 1-2-1 weighting. Any cooling in the years after AD 1783 probably were due to the Laki eruption and not the Asama eruption[64]. Composite of changes in northern hemisphere temperature for the four years before and after major eruptions of the last two centuries (bottom). Reprinted from *Quaternary Science Reviews*, **19**, Use of paleo-records in determining variability within the volcanism-climate system, 417-438, 2000, with permission from Elsevier Science. Initial permission from American Meteorological Society for top figure and from Elsevier Science for bottom figure.

Although cooling is obvious following certain eruptions, there is regional variability in the timing and amount of cooling, particularly given an equatorial vs. a high-latitude eruption, as discussed above. By looking at the magnitude and timing of volcanically induced temperature depression over the last roughly 200 years at low, mid-, and high latitudes as well as in the Northern Hemisphere as a whole, it was found that peaks in the cooling from an equatorial eruption were cyclical, coinciding with the hemispheric distribution of the aerosols seasonally[36]. Peak cooling occurs at higher latitudes during the summer of the first and second years following an eruption as stratospheric aerosols are dispersed from the tropics into higher latitudes each spring. By comparison, the Northern Hemisphere would feel a quicker and more pronounced cooling immediately following a high northern-latitude eruption compared to that from an equatorial eruption.

The complexity of the climatic response to an eruption also has been shown by evaluation of seasonal temperature changes following the Pinatubo and El Chichón eruptions[37,38]. Following both eruptions, Eurasian winters were often warmer than average. The perturbation to the latitudinal energy budget from the presence of stratospheric aerosols in the tropics leads to frequent advection of warm air into Eurasia, although this same area, as well as the northeastern U.S., exhibits much colder than average summers following a major eruption. These conditions seem to occur regardless of the location of the eruption.

An additional factor that can modify the climatic impact of an eruption is its coincidence with an El Niño event. This would diminish the climatic cooling as observed following the El Chichón eruption in AD 1982[39,40]. For example, both Northern and Southern Hemisphere surface temperatures were surprisingly warm after El Chichón in comparison to the cooling following the AD 1963 Agung event[41]. The amount of sulfur produced by the El Chichón eruption is estimated to be slightly less than or possibly greater than that from Agung[42]. Identifying the cause of the lack of cooling following the El Chichón eruption was undertaken by evaluating the impact of warmer sea-surface temperatures on global temperature. By removing the El Niño impact, it was found that the magnitude of cooling associated with El Chichón was about 0.33˚C. This same exercise showed that the magnitude of cooling following other eruptions in the last 2 centuries was lessened to some degree by a subsequent El Niño. However, there is no convincing evidence that a volcanic eruption may induce an El Niño[13,43].

## Beyond Instrumental Records: Proxy Records

Instrumental meteorological records in combination with satellite data have provided much insight into the climatic impact of volcanism. Unfortunately, these conclusions are based on a limited sampling of eruption types during the 200+ years of instrumental records and especially during the 20–25 years of satellite availability. Furthermore, we are not able to look at how a specific type of eruption would impact climate if it occurred when the Earth's climate was in one particular mode vs. the contrasting mode (such as glacial vs. interglacial conditions, to use the extremes). Consequently, it is necessary to look into the past. In doing this, it is necessary to take a multidisciplinary approach in evaluating the climatic impact of past volcanism (as discussed in detail in[8]).

There are many types of proxy data that may be used in evaluating volcanism beyond the instrumental record (as summarized in[8]), but three types of data probably have provided the most significant information used in this process. They are the volcanic records available in ice cores, tree-ring chronologies, and geological deposits of past eruptions. Examples of the type of relevant data available in these records follow.

Ice cores arguably provide the best chronology of climatically effective volcanism because they record the deposition of the stratospheric aerosols directly responsible for the climate forcing, and their records are continuous, highly resolved (subseasonal to annual to decadal), as well as lengthy (up to 100,000+ years). After migration of the stratospheric cloud from the latitude of the eruption to the polar regions, sedimentation of these aerosols into the troposphere results in the eventual scavenging by snowfall and deposition on the glacier surface. Freshly fallen snow is then compacted by subsequent snowfalls, eventually forming glacial ice. The temporal resolution available in a single core and the total length of discernible record are functions of accumulation rates, with relatively high accumulation rates providing great detail over a shorter period of time (e.g., summit of Greenland) compared to relatively low accumulation rates that provide less-resolved records over a longer time period (e.g., East Antarctica plateau).

The ice-core parameters that document the presence of volcanic aerosols are: $SO_4^{2-}$ (a direct measurement of the $H_2SO_4$ component of the record, such as[44,45,46]) and electrical properties of the ice, such as conductivity (i.e., ECM, which measures total acids, as in[47]) and the dipolar method (i.e., DEP, which measures total salts, as in[7]). Evidence of the deposition of volcanic acids is found in the very large spikes in these glaciochemical records that were found to correspond to the known record of recent volcanism given the annual dating of most ice-core records (Fig. 3). More recently, records of volcanic acid deposition have been supplemented with the analysis of volcanic glass found in the same layers of the ice core, thereby verifying the source eruption or providing evidence of the contribution of multiple eruptions to the acidity signals[48].

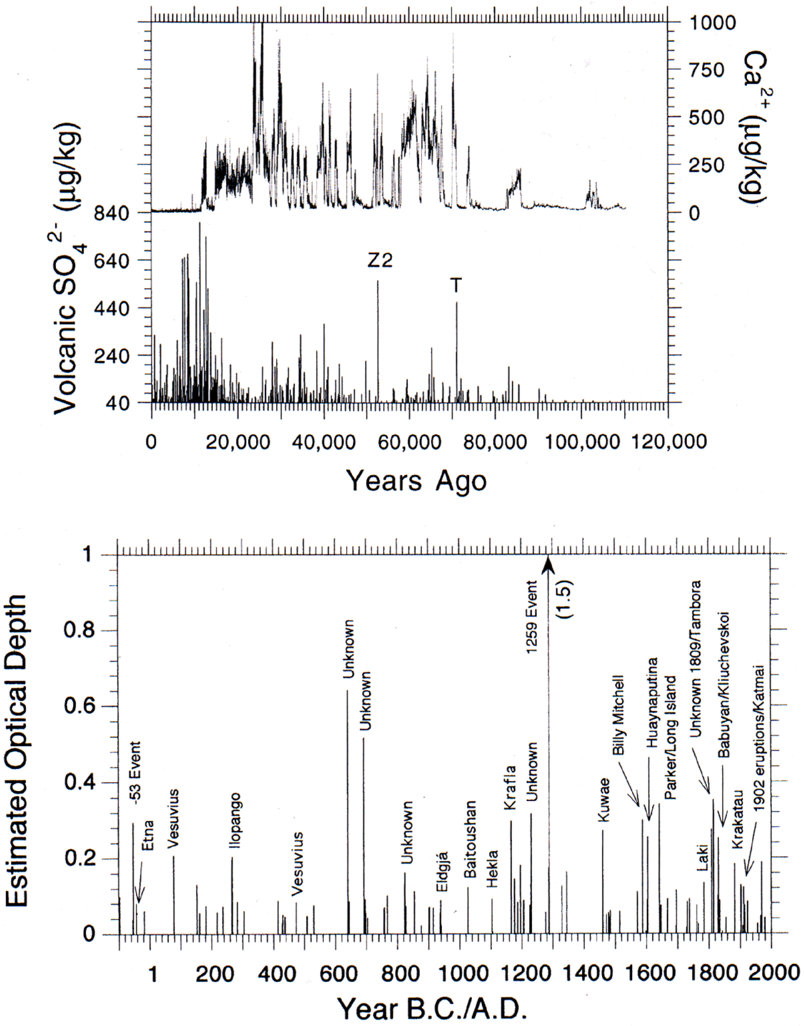

**FIGURE 3.** The 110,000-year volcanic $SO_4^{2-}$ record from the GISP2 ice core compared to the $Ca^{2+}$ record (top). $Ca^{2+}$ is a proxy for climatic conditions with high values corresponding to cold conditions and low values corresponding to warm conditions. Signals associated with the Z-2 ash equivalent found in North Atlantic sediment cores (Z) and for the Toba (T) eruption are noted. See [8, 65] for details. Intermediate estimates of the optical depth ($\tau$) produced by volcanic eruptions over the last 2100 years as recorded in the GISP2 ice core (bottom). See [8] and the original discussion of the technique used to derive these values[66]. Reprinted from *Quaternary Science Reviews*, **19**, Use of paleo-records in determining variability within the volcanism-climate system, 417-438, 2000, with permission from Elsevier Science. Initial permission from Amcerican Geophysical Union.

Tree-ring records provide an excellent link to the climatic impact of past eruptions because of the excellent chronology developed (i.e., to within a year) and the correlations made between various tree-ring characteristics[8] and known volcanic eruptions (Fig. 4). For example, an excellent correlation between narrow rings and/or low densities with summer temperatures verifies the forcing factor responsible for these phenomena. Transfer functions have been developed in several studies[49,50] to quantify the cooling in summers following volcanic eruptions. Moreover, the strongest signals found in northern boreal forest composite records[51] match well with the GISP2 (Greenland Ice Sheet Project) ice-core record, particularly the very large Kuwae (~AD 1453), Huaynaputina (AD 1600), Parker (AD 1641), and Tambora (AD 1815) eruptions (Figs. 3 and 4). The good agreement between these two recently developed, highly resolved volcanic records is encouraging, especially when the chronology of volcanic signals developed in ice-core and tree-ring records from just 10+ years ago did not match up this well[52].

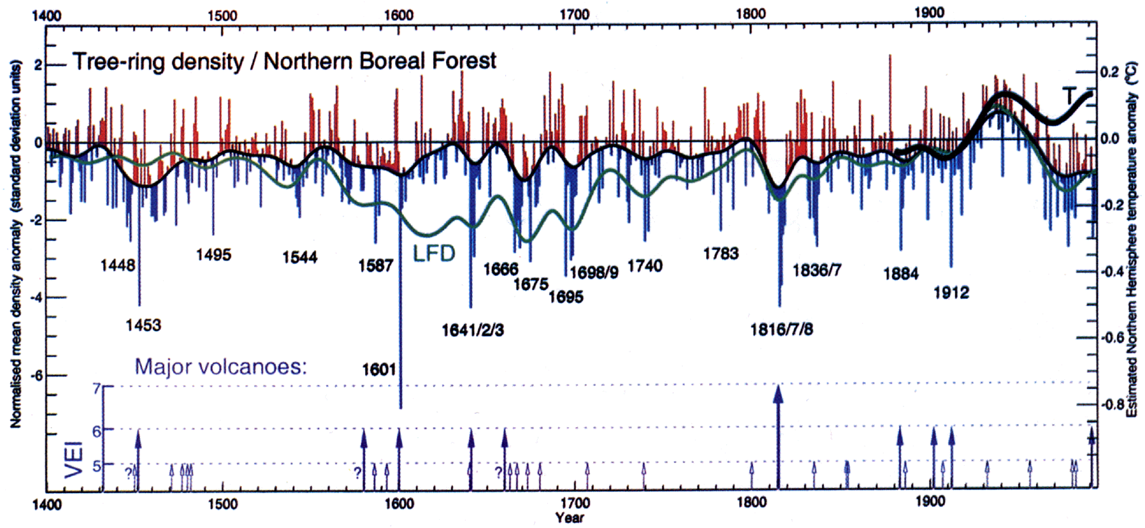

**FIGURE 4.** Growing season temperature changes in the northern hemisphere (T) as associated with volcanic eruptions over the last 600 years based on yearly average of maximum tree-density (histogram) [51]. LFD indicates low-frequency density changes. See [51] and references therein for details on use of LFD. Large volcanic eruptions indicated by the Volcanic Explosivity Index (VEI)[66]. Reprinted from *Quaternary Science Reviews*, **19**, Annual climate variability in the Holocene: Interpreting the message of ancient trees, 87-105, 2000, with permission from Elsevier Science. Initial permission from *Nature*.

Volcanological data, particularly those obtained from field studies, provide much critical information necessary to evaluate the climatic impact of an eruption[8]. However, the estimate of the amount of sulfur degassed via the petrological technique provides the most relevant data (such as[53]). This technique compares the sulfur content of inclusions in a phenocryst, thought to represent the initial sulfur composition of the magma, with that found in matrix glass. The difference is thought to be the amount degassed into the atmosphere and when combined with estimates of the volume erupted can lead to estimates of the mass of $SO_2$ and $H_2SO_4$ produced by the eruption. The technique also estimates the amount of $Cl^-$ (HCl) and $F^-$ (HF) produced. However, there are some problems with the technique, as estimates of the amount of sulfur produced by the El Chichón eruption by the petrologic technique were much lower than those estimated from satellite data[54]. Refinements in the technique now appear to be able to provide estimates of sulfur degassing that closely match other independent estimates[55].

## Multiple Eruptions and Mega-Eruptions

A major contribution made by evaluating the relationship between climate and volcanic eruptions has been the ability to evaluate more completely how multiple eruptions closely spaced in time and how mega-eruptions, which have not been felt by society in historical time, may impact climate. Lengthy and continuous records of both the chronology of volcanism and the climatic impact (via stratospheric mass loading, optical depth measurements, and temperature changes) are essential to modeling experiments used to determine which naturally occurring forcing factors have played a role in recent climatic change. Once natural variability can be isolated, the contribution of humans to recent climatic change can be better assessed.

The ice-core and tree-ring chronologies shown here indicate that there were several periods, particularly in the 1600s and 1800s, that were marked by significant episodes of volcanism (Figs. 3 and 4). These are periods of significant Northern Hemisphere cooling during the Little Ice Age. Modeling experiments verify that volcanism, together with solar variability, has played a significant role in forcing climate over the last 300–400 years both globally[56] and particularly in the Arctic[57]. These more recent findings verify earlier work suggesting that Little Ice Age climate was, in part, forced by volcanism[58]. The long time series of volcanism from these proxy records also has provided necessary data to evaluate how volcanism has forced climate over even longer time periods. It has been found that as much as 41–64% of preanthropogenic (pre-1850) decadal-scale temperature variability over the last 1000 years was due to changes in solar irradiance or volcanism[59]. Of that variability, volcanism accounts for 22–23% of the temperature change. However, increased volcanism over the last 600 years (relative to the prior 400 years, Fig. 3) was responsible for 41–49% of the temperature variability between 1400 and 1850 (i.e., during the Little Ice Age)[60].

Light also has been shed on the climatic impact of mega-eruptions through the use of these proxy data. The eruption of Toba, Sumatra, that occurred about 75,000 years ago produced 2500–3000 $km^3$ of magma (dense rock equivalent), almost two orders of magnitude greater than that produced by the largest known recent historical eruption (Tambora)[59]. The $1 \times 10^{15}$ to $1 \times 10^{16}$ g $H_2SO_4$ that may have been produced by the Toba eruption[60] would have been enough to affect climate drastically. Even the minimum estimate of stratospheric loading ($1 \times 10^{15}$) would have reduced sunlight to about one tenth of a cloudless day at high noon[42], a scenario that has significant implications, particularly for inducing the period of glaciation around that time period. The potential regional to possibly hemispheric annual cooling of 3–5˚C following the Toba eruption together with the possibility that summer cooling on the order of ≥10˚C occurred is enough to lead to increased perennial snowcover and sea-ice extent. In general, these factors easily could have accelerated the global cooling under way at that time from changing orbital parameters[61].

Sampling of the highly resolved GISP2 ice core[62] showed that the Toba eruption occurred at the beginning of a 1000-year stadial (cold) event and not at the beginning of the 10,000-year glacial event (i.e., isotopic stage 4), thereby eliminating the possibility that Toba could have initiated the glacial event (Fig. 5). However, there still is the question as to whether or not the eruption could have initiated the 1000-year stadial (cold) event indicated by the ice-core parameters. Annual sampling of the Toba section in the GISP2 core signal indicates that high sulfur loading from a maximum of $2–4 \times 10^{15}$ g $H_2SO_4$ may have existed in the stratosphere for a period of up to 7 years. Modeling work[63] similarly suggested that the residence time of the aerosols could have been up to 7 years. These were the first results to imply that the aerosols from a volcanic eruption may have stayed aloft for such a lengthy period of time. These findings may mean that various feedback mechanisms could begin, such as cooling of sea-surface temperatures, which would then lead to a lengthy period of global cooling. In general, there seems to be several centuries of enhanced cooling following Toba that do not appear in other stadial events during the last glacial period. Consequently, Toba may have enhanced cooling through various feedback mechanisms on century time scales. Such an eruption and the resulting feedbacks and climatic change would have extremely serious social and economic repercussions should it happen today.

## CONCLUSIONS

Sulfur-rich gases produced by a volcanic eruption oxidize to $H_2SO_4$ aerosols in the stratosphere and both absorb and reflect incoming solar radiation. This scattering process warms the stratosphere but cools the Earth's surface. Silicate material produced during the eruption does not remain aloft for a long enough period of time to have a widespread impact on climate. Moreover, the eruptive plume needs to penetrate the tropopause to prevent the washout of aerosols by precipitation. Plinian eruptions are often able to penetrate the tropopause, although buoyant plumes above fire fountains in effusive eruptions also can reach the stratosphere. As a result, climatically effective eruptions are sulfur-rich and explosive enough to inject material into the stratosphere. Eruptions able to affect climate need to eject at least 1 Mt of $SO_2$ into the stratosphere, which ultimately would produce about 12.5 Mt of $H_2SO_4$ aerosols. The resulting

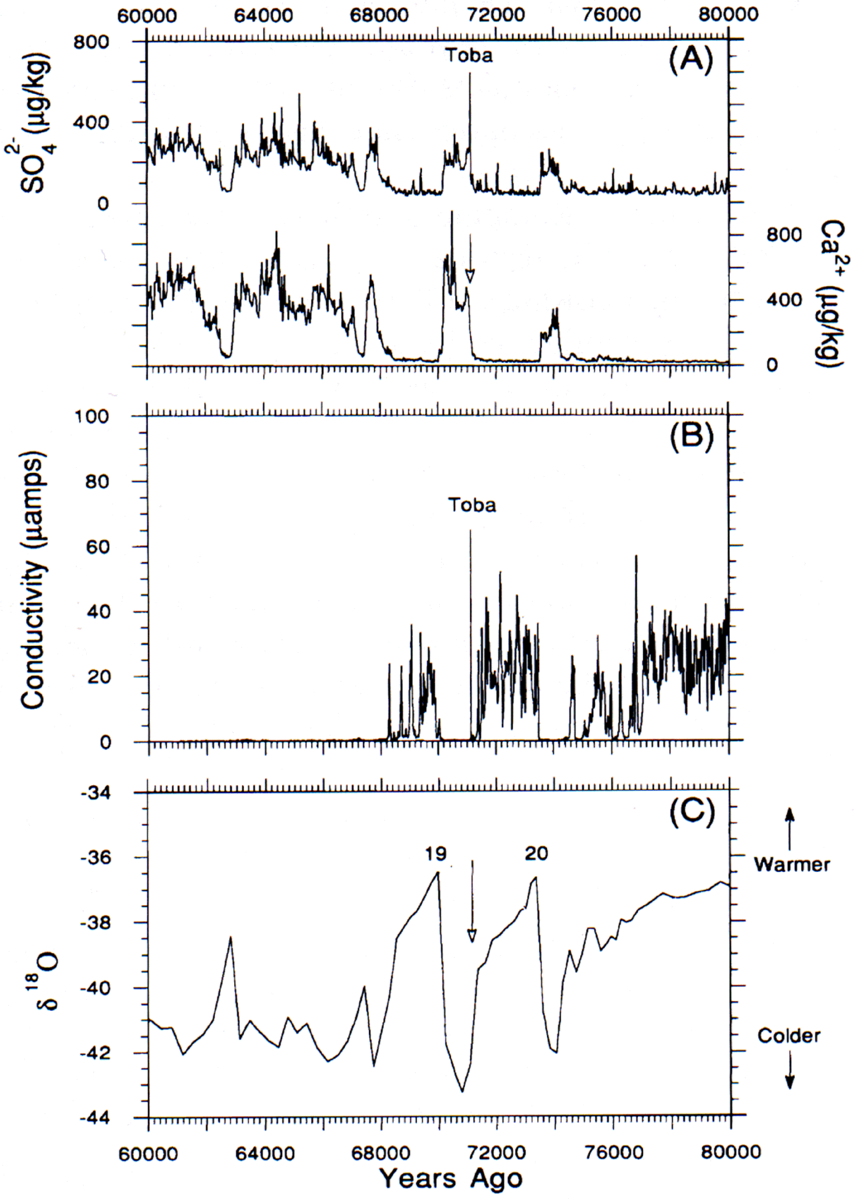

**FIGURE 5.** Time series of $SO_4^{2-}$ (A) and $Ca^{2+}$(A), electrical conductivity (B), and $\delta^{18}O$ (C) between 60,000 and 80,000 years ago showing the signal associated with the Toba (arrow) mega-eruption in the GISP2 ice core. 19 and 20 represent interstadial (warm) events used as climatic markers in the GISP2 core. A stadial (cold) event separates these interstadial events. The glacial period begins about 68,000 years ago. See text and [61] for details. Reprinted from *Quaternary Science Reviews*, 19, Use of paleo-records in determining variability within the volcanism-climate system, 417-438, 2000, with permission from Elsevier Science. Initial permission from American Geophysical Union.

optical depth ($\tau$) of such an eruption would be about 0.1, high enough to cool the Earth's surface. To have a global impact, the eruption must originate in the equatorial region; mid- to high-latitude eruptions will impact only the hemisphere of origin. On the other hand, less explosive eruptions may reach the stratosphere in mid- to high latitudes, particularly during the winter, because of the lower tropopause (8–10 km) than that in equatorial regions (15 km).

Volcanic eruptions are clearly able to force rapid climatic change (the type of change that has significant impact on humans) and to change climate on decadal to centennial time scales.

Individual eruptions that meet the necessary criteria may cool global or hemispheric climate by 0.2–0.3˚C for several years after the eruption, with maximum cooling occurring during the first 2 years following the eruption. On the other hand, multiple eruptions closely spaced in time and especially mega-eruptions have the potential to impact social and economic systems on decadal to possibly centennial scales, respectively. Adding to the complexity of the volcanism-climate system is the fact that the resulting impact of an eruption is influenced by the climatic mode in existence at the time of the eruption. An eruption during a much warmer mode may have a more limited impact, as would happen with the simultaneous occurrence of an eruption and an El Niño event. An eruption that occurs during a cooler climatic mode may enhance or extend those cooler conditions. The latitude of the eruption is also critical in the timing of the maximum cooling of the eruption. The nature of the climatic change will also vary spatially. Certain areas will feel maximum cooling during the summer or summers following the eruption, while these same areas or other areas may experience an increase in temperature, particularly during the winter. Thus, volcanic eruptions have been a major forcing factor of climate, and they will continue to do so in the future.

## ACKNOWLEDGMENTS

I thank the many individuals with whom I have collaborated in my research on the volcanism-climate system, particularly S. Self, A. Robock, K. Hirschboeck, K. Pang, and R. Keen. Special appreciation is given to members of the ice-core community for providing glaciochemical and other types of data from many of the ice cores analyzed and especially those from the GISP2 core. Their help in evaluating these data sets is greatly appreciated as well. The primary individuals are P. Mayewski, M. Twickler, S. Whitlow, D. Meeker, and J. Dibb. The list is too lengthy to mention the many others in the ice-core community who have helped in different ways, but my thanks is noted. Fruitful discussions with M. Rampino, D. Pyle, S. Carey, M. Baillie, H. Sigurdsson, and K. Briffa among others have helped in evaluating and compiling information eventually used in this summary. Support for much of my work has come from the Office of Polar Programs, U.S. National Science Foundation with additional support used to compile the multidisciplinary records of several past eruptions provided by the Division of Atmospheric Sciences, U.S. National Science Foundation.

## References

1. Franklin, B. (1784) Meteorological imaginations and conjectures. *Manchester Lit. Philos. Soc. Mem. Proc.* **2**, 22.
2. Sigurdsson, H. (1982) Volcanic pollution and climate: the 1783 Laki eruption. *EOS* **63**, 601–602.
3. Stommel, H. and Stommel, E. (1983) *Volcano Weather*. Seven Seas Press, Newport, RI.
4. Harrington, C.R., Ed. (1992) *The Year Without a Summer? World Climate in 1816*. Canadian Museum of Nature, Ottawa.
5. Chenoweth, M. (2001) Two major volcanic cooling episodes derived from global marine air temperature, AD 1807-1827. *Geophys. Res. Lett.* **28**, 2963–2966.
6. Dai, J., Mosley-Thompson, E., and Thompson, L.G. (1991) Ice core evidence for an explosive tropical volcanic eruption 6 years preceding Tambora. *J. Geophys. Res.* **96**, 17,361–17,366.
7. Moore, J.C., Narita, H., and Maeno, N. (1991) A continuous 770-year record of volcanic activity from East Antarctica. *J. Geophys. Res.* **96**, 17,353–17,359.
8. Zielinski, G.A. (2000) Use of paleo-records in determining variability within the volcanism-climate system. *Quat. Sci. Rev.* **19**, 417–438.
9. Lamb, H.H. (1970) Volcanic dust in the atmosphere with a chronology and assessment of its meteorological significance. *Philos. Trans. R. Soc. London* **266**, 425–533.
10. Toon, O.B. (1982) Volcanoes and climate. In *Atmospheric Effects and Potential Climatic Impact of the 1980 Eruptions of Mount St. Helens.* Deepak, A., Ed. NASA Conference Publication 2240, pp. 15–36.
11. Ellsaeser, H.W. (1983) Isolating the Climatologic Effects of Volcanoes. Report UCRl-8916. Lawrence Livermore National Laboratory, Livermore, CA.
12. Kondratyev, K.Y. and Galindo, I. (1997) *Volcanic Activity and Climate*. A. Deepak Publishing, Hampton, VA.
13. Robock, A. (2000) Volcanic eruptions and climate. *Rev. Geophys.* **38**, 191–219.

14. Hofmann, D.J. (1987) Perturbations to the global atmosphere associated with the El Chichon volcanic eruption of 1982. *Rev. Geophys.* **25**, 743–759.
15. McCormick, M.P., Thomason, L.W., and Trepte, C.R. (1995) Atmospheric effects of the Mt. Pinatubo eruption. *Nature* **373**, 399–404.
16. Pollack, J.B., Toon, O.B., Sagan, C., Summers, A., Baldwin, B., and Van Camp, W. (1976) Volcanic explosions and climatic change: a theoretical assessment. *J. Geophys. Res.* **81**, 1071–1083.
17. Robock, A. and Mass, C. (1982) The Mount St. Helens volcanic eruption of 18 May 1980: large short-term surface temperature effects. *Science* **216**, 628–630.
18. Self, S., Zhao, J.-X., Holasek, R.E., Torres, R.C., and King, A.J. (1996) The atmospheric impact of the 1991 Mount Pinatubo eruption. In *Fire and Mud: Eruptions and Lahars of Mount Pinatubo, Philippines.* Newhall, C.G. and Punongbayan, R.S., Eds. University of Washington Press, Seattle, pp. 1089–1115.
19. Bluth, G.J.S., Doiron, S.D., Schnetzler, C.C., Krueger, A.J., and Walter, L.S. (1992) Global tracking of the $SO_2$ clouds from the June, 1991 Mount Pinatubo eruptions. *Geophys. Res. Lett.* **19**, 151–154.
20. Tabazadeh, A. and Turco, R.P. (1993) Stratospheric chlorine injection by volcanic eruptions: HCl scavenging and implications for ozone. *Science* **260**, 1082–1086.
21. Rose, W.I., Jr. (1977) Scavenging of volcanic aerosol by ash: atmospheric and volcanologic implications. *Geology* **5**, 621–624.
22. Lyons, W.B., Mayewski, P.A., Spencer, M.J., Twickler, M.S., and Graedel, T.E. (1990) A northern hemisphere volcanic chemistry (1869–1984) and climatic implications using a South Greenland ice core. *Ann. Glaciol.* **14**, 176–182.
23. De Angelis, M. and Legrand, M. (1994) Origins and variations of fluoride in Greenland precipitation. *J. Geophys. Res.* **99**, 1157–1172.
24. Stothers, R.B. (1996) Major optical depth perturbations to the stratosphere from volcanic eruptions: the pyrheliometric period, 1881-1969. *J. Geophys. Res.* **101**, 3901–3920.
25. Stothers, R.B. (1984) Mystery cloud of AD 536. *Nature* **307**, 344–345.
26. Bluth, G.J.S., Schnetzler, C.C., Krueger, D.A.J., and Walter, L.S. (1993) The contribution of explosive volcanism to global sulfur dioxide concentrations. *Nature* **366**, 327–330.
27. Simarski, L.T. (1992) Volcanism and climate change. American Geophysical Union Special Report, Washington, D.C.
28. Stothers, R.B., Wolff, J.A., Self, S., and Rampino, M.R. (1986) Basaltic fissure eruptions, plume heights, and atmospheric aerosols. *Geophys. Res. Lett.* **13**, 725–728.
29. Thordarson, T. and Self, S. (1993) The Laki (Skaftar Fires) and Grimsvotn eruptions in 1783-1785. *Bull. Volcanol.* **55**, 233–263.
30. Stothers, R.B. (1996) The great dry fog of 1783. *Climatic Change* **32**, 79–89.
31. Gerlach, T.M., Westrich, H.R., Casadevall, T.J., and Finnegan, D.L. (1994) Vapor saturation and accumulation in magmas of the 1989-1990 eruption of Redoubt Volcano, Alaska. *J. Volcanol. Geotherm. Res.* **62**, 317–337.
32. Sato, M., Hansen, J.E., McCormick, M.P., and Pollack, J.B. (1993) Stratospheric aerosol optical depths, 1850–1990. *J. Geophys. Res.* **98**, 22,987–22,994.
33. Mills, M.J. (2000) Volcanic aerosol and global atmospheric effects. In *Encyclopedia of Volcanoes.* H. Sigurdsson, Ed. Academic Press, San Diego.
34. Angell, J.K. and Korshover, J. (1985) Surface temperature changes following six major volcanic episodes between 1780 and 1980. *J. Climate Appl. Meteorol.* **24**, 937–951.
35. Self, S., Rampino, M., and Barbera, J.J. (1981) The possible effects of large 19th and 20th century volcanic eruptions on zonal and hemispheric surface temperatures. *J. Volcanol. Geotherm. Res.* **11**, 41–60.
36. Bradley, R.S. (1988) The explosive volcanic eruption signal in northern hemisphere continental temperature records. *Climatic Change* **12**, 221–243.
37. Robock, A. and Mao, J. (1992) Winter warming from large volcanic eruptions. *Geophys. Res. Lett.* **12**, 2405–2408.
38. Groisman, P.Y. (1992) Possible regional climate consequences of the Pinatubo eruption. *Geophys. Res. Lett.* **19**, 1603–1606.
39. Mass, C.F. and Portman, D.A. (1989) Major volcanic eruptions and climate: a critical assessment. *J. Climatol.* **2**, 566–593.
40. Portman, D.A. and Gutzler, D.S. (1996) Explosive volcanic eruptions, the El Niño-Southern Oscillation, and U.S. climate variability. *J. Climate* **9**, 17–33.
41. Angell, J.K. (1988) Impact of El Niño on the delineation of tropospheric cooling due to volcanic eruptions. *J. Geophys. Res.* **93**, 3697–3704.
42. Rampino, M.R., Self, S., and Stothers, R.B. (1988) Volcanic winters. *Annu. Rev. Earth Planetary Sci.* **16**, 73–99.
43. Self, S., Rampino, M.R., Zhao, J., and Katz, M.G. (1997) Volcanic aerosol perturbations and strong El Niño events: no general correlation. *Geophys. Res. Lett.* **24**, 1247–1250.
44. Legrand, M. and Delmas, R.J. (1987) A 220-year continuous record of volcanic $H_2SO_4$ in the Antarctic Ice Sheet. *Nature* **327**, 671–676.

45. Mayewski, P.A., Holdsworth, G., Spencer, M.J., Whitlow, S., Twickler, M., Morrison, M.C., Ferland, K.K., and Meeker, L.D. (1993) Ice core sulfate from three northern hemisphere sites: source and temperature forcing implications. *Atmos. Environ.* Part A, **27**, 2915–2919.
46. Zielinski, G.A., Mayewski, P.A., Meeker, L.D., Whitlow, S., Twickler, M.S., Morrison, M., Meese, D., Alley, R.B., and Gow, A.J. (1994) Record of volcanism since 7000 B.C. from the GISP2 Greenland ice core and implications for the volcano-climate system. *Science* **264**, 948–952.
47. Hammer, C.U., Clausen, H.B., and Dansgaard, W. (1980) Greenland ice sheet evidence of post-glacial volcanism and its climatic impact. *Nature* **288**, 230–235.
48. Zielinski, G.A., Mayewski, P.A., Meeker, L.D., Grönvold, K., Germani, M.S., Whitlow, S., Twickler, M.S., and Taylor, K. (1997) Volcanic aerosol records and tephrochronology of the Summit, Greenland, ice cores. *J. Geophys. Res.* **102**, 26,625–26,640.
49. Jones, P.D., Briffa, K.R., and Schweingruber, F.H. (1995) Tree-ring evidence of the widespread effects of explosive volcanic eruptions. *Geophys. Res. Lett.* **22**, 1333–1336.
50. Briffa, K.R., Jones, P.D., Schweingruber, F.H., and Osborn, T.J. (1998) Influence of volcanic eruptions on Northern Hemisphere summer temperatures over the past 600 years. *Nature* **393**, 450–455.
51. Briffa, K.R. (2000) Annual climate variability in the Holocene: interpreting the message of ancient trees. *Quat. Sci. Rev.* **19**, 87–105.
52. Pyle, D.M. (1992) On the "climatic effectiveness" of volcanic eruptions. *Quat. Res.* **37**, 125–129.
53. Devine, J.D., Sigurdsson, H., Davis, A.N., and Self, S. (1984) Estimates of sulfur and chlorine yield to the atmosphere from volcanic eruptions and potential climatic effects. *J. Geophys. Res.* **89**, 6309–6325.
54. Luhr, J.F., Carmichael, I.S.E., and Varekamp, J.C. (1984) The 1982 eruptions of El Chichón Volcano, Chiapas, Mexico: mineralogy and petrology of the anhydrite-bearing pumices. *J. Volcanol. Geotherm. Res.* **23**, 69–108.
55. Self, S., and King, A.J. (1996) Petrology and sulfur and chlorine emissions of the 1963 eruption of Gunung Agung, Bali, Indonesia. *Bull. Volcanol.* **58**, 263–285.
56. Bertrand, C., van Ypersele, J.-P., and Berger, A. (1999) Volcanic and solar impacts on climate since 1700. *Climate Dynam.* **15**, 355–367.
57. Overpeck, J., Hughen, K., Hardy, D., Bradley, R., Case, R., Douglas, M., Finney, B., Gajewski, K., Jacoby, G., Jennings, A., Lamoureux, S., Lasca, A., MacDonald, G., Moore, J., Retelle, M., Smith, S., Wolfe, A., and Zielinski, G. (1997) Arctic environmental change of the last four centuries. *Science* **278**, 1251–1256.
58. Robock, A. (1979) The "Little Ice Age": Northern Hemisphere average observations and model calculations. *Science* **206**, 1402–1404.
59. Crowley, T.J. (2000) Causes of climate change over the past 1000 years. *Science* **14**, 270–277.
60. Chesner, C.A., Rose, W.I., Deino, A., Drake, R., and Westgate, J.A. (1991) Eruptive history of Earth's largest Quaternary caldera (Toba, Indonesia) clarified. *Geology* **19**, 200–203.
61. Rampino, M.R. and Self, S. (1992) Volcanic winter and accelerated glaciation following the Toba super-eruption. *Nature* **359**, 50–52.
62. Zielinski, G.A., Mayewski, P.A., Meeker, L.D., Whitlow, S., Twickler, M., and Taylor, K. (1996) Potential atmospheric impact of the Toba mega-eruption ~71,000 years ago. *Geophys. Res. Lett.* **23**, 837–840.
63. Bekki, S., Pyle, J.A., Zhong, W., Toumi, R., Haigh, J.D., and Pyle, D.M. (1996) The role of microphysical and chemical processes in prolonging the climate forcing of the Toba eruption. *Geophys. Res. Lett.* **23**, 2669–2672.
64. Zielinski, G.A., Fiacco, R.J., Whitlow, S., Twickler, M.S., Germani, M.S., Endo, K., and Yasui, M. (1994) Climatic impact of the AD 1783 eruption of Asama (Japan) was minimal: evidence from the GISP2 ice core. *Geophys. Res. Lett.* **21**, 2365–2368.
65. Zielinski, G.A., Mayewski, P.A., Meeker, L.D., Whitlow, S., and Twickler, M. (1996) A 110,000-year record of explosive volcanism from the GISP2 (Greenland) ice core. *Quat. Res.* **45**, 109–118.
66. Zielinski, G.A. (1995) Stratospheric loading and optical depth estimates of explosive volcanism over the last 2100 years derived from the GISP2 Greenland ice core. *J. Geophys. Res.* **100**, 20,937–20,955.
67. Newhall, C.G. and Self, S. (1982) The Volcanic Explosivity Index (VEI): an estimate of explosive magnitude for historical volcanism. *J. Geophys. Res.* **87**, 1231–1238.
